# Antitumor Therapy under Hypoxic Microenvironment by the Combination of 2-Methoxyestradiol and Sodium Dichloroacetate on Human Non-Small-Cell Lung Cancer

**DOI:** 10.1155/2020/3176375

**Published:** 2020-10-23

**Authors:** Yair Romero, Manuel Castillejos-López, Susana Romero-García, Alfonso Salgado Aguayo, Iliana Herrera, Misael O. Garcia-Martin, Luz Maria Torres-Espíndola, Maria Cristina Negrete-García, Angel Camarena Olvera, Juan Carlos Huerta-Cruz, Rafael Velázquez-Cruz, José Cisneros, Edgar Flores Soto, Héctor Solís-Chagoyán, Criselda Mendoza-Milla, Carlos Cabello-Gutiérrez, Víctor Ruiz, Arnoldo Aquino-Gálvez

**Affiliations:** ^1^Facultad de Ciencias, Universidad Nacional Autónoma de México, CDMX, Mexico; ^2^Instituto Nacional de Enfermedades Respiratorias “Ismael Cosío Villegas”, CDMX, Mexico; ^3^Instituto Nacional de Pediatría, CDMX, Mexico; ^4^Instituto Nacional de Medicina Genómica, CDMX, Mexico; ^5^Facultad de Medicina, Universidad Nacional Autónoma México, CDMX, Mexico; ^6^Instituto Nacional de Psiquiatría “Ramón de la Fuente Muñiz”, CDMX, Mexico

## Abstract

A hypoxic microenvironment is a hallmark in different types of tumors; this phenomenon participates in a metabolic alteration that confers resistance to treatments. Because of this, it was proposed that a combination of 2-methoxyestradiol (2-ME) and sodium dichloroacetate (DCA) could reduce this alteration, preventing proliferation through the reactivation of aerobic metabolism in lung adenocarcinoma cell line (A549). A549 cells were cultured in a hypoxic chamber at 1% O_2_ for 72 hours to determine the effect of this combination on growth, migration, and expression of hypoxia-inducible factors (HIFs) by immunofluorescence. The effect in the metabolism was evaluated by the determination of glucose/glutamine consumption and the lactate/glutamate production. The treatment of 2-ME (10 *μ*M) in combination with DCA (40 mM) under hypoxic conditions showed an inhibitory effect on growth and migration. Notably, this reduction could be attributed to 2-ME, while DCA had a predominant effect on metabolic activity. Moreover, this combination decreases the signaling of HIF-3*α* and partially HIF-1*α* but not HIF-2*α*. The results of this study highlight the antitumor activity of the combination of 2-ME 10 *μ*l/DCA 40 mM, even in hypoxic conditions.

## 1. Introduction

Hypoxia is a recurrent phenomenon in tumors and induces alterations that drive its progression [1, 2]. Adaptations to hypoxia are mainly controlled by hypoxia-inducible factors (HIFs); HIFs are dimeric transcription factors that involve a HIF-alpha subunits (HIF-1*α*, HIF-2*α*, or HIF-3*α*) and a beta subunit. Alpha subunits are degraded by the ubiquitin-proteasome system in an oxygen-dependent manner. The accumulation and translocation of HIF dimers to the nucleus activate genes related to glycolysis, proliferation, survival, and angiogenesis, invasion, and metastasis [3, 4].

Although metabolic changes in cancer were described almost a century ago by Otto Warburg, the metabolism in cancer biology is not fully understood [5, 6]. Therefore, this study relies on the metabolic alterations induced by hypoxia and trying to revert them with a combination of 2-methoxyestradiol (2-ME) and sodium dichloroacetate (DCA) drugs that have a potential role decreasing mechanisms and molecules important in tumor development. 2-ME is a natural metabolite of endogenous estrogens 17b-estradiol and 2-hydroxiestradiol that has been reported to have antitumor activity, because it reduces growth, angiogenesis, and HIF-1*α* signaling *in vivo* and *in vitro* [7, 8]. Among numerous drugs that target metabolism, dichloroacetate (DCA) has shown excellent potential because of its positive contribution to cancer treatment; DCA is a small molecule that has been used to treat lactic acidosis by the inhibition of pyruvate kinase (PDK) and to reactivate aerobic metabolism [9]. Thus, a combination of these drugs might be able to help redirect an aerobic one in a lung adenocarcinoma cell line.

The objective of this work was to evaluate if the combination of 2-ME and DCA under hypoxia had an effect on the growth, migration, glucose/glutamine consumption, and expression of alpha subunits of HIF-1, 2, and 3.

## 2. Materials and Methods

### 2.1. Ethics

The protocol was approved by the Ethics and Research Committees of the Instituto Nacional de Enfermedades Respiratorias (INER No. B17-16).

### 2.2. Reagents and Cells

A stock solution of 2-methoxyestradiol (2-ME) (Sigma-Aldrich) was prepared in DMSO. Sodium dichloroacetate (DCA) (Sigma-Aldrich) was diluted at 40 mM in Dulbecco's modified Eagle's medium (DMEM) with 10% FBS. A549 cell line was purchased from the American Type Culture Collection (ATTC).

### 2.3. Hypoxia

Culture flasks were placed in a humidified chamber MIC-101 (Billups-Rothenberg, Inc., CA.) and maintained at 1% of O_2_. A mixture of nitrogen and carbon dioxide gas was used to displace the oxygen; the oxygen concentration was measured by an oxygen analyzer (Teledyne Analytical Instruments) with a sensor OOM105 (EnviteC-Wismar).

### 2.4. Cell Growth Assay

Cell growth assay was performed as previously described (8). Briefly, cells were fixed with 1% glutaraldehyde at room temperature and incubated with crystal violet solution (N-hexa-methylpararosaniline, Sigma-Aldrich). Absorbance was measured according to the manufacturer's recommendations in a plate reader (Molecular Devices, CA, USA).

### 2.5. Wound Healing Assay

8 × 10^5^ cells were plated in 12-well cell culture dishes and preincubated with mitomycin C (Sigma-Aldrich) at a concentration of 2 *μ*g/ml. An artificial wound was created by disrupting the monolayer with a sterile 10 *μ*l plastic pipette tip. Cells were washed with PBS, and photographs were taken immediately after scratching (baseline). Consecutive photographs were taken using an EVOS XL microscope (Life Technologies). Area was quantified using ImageJ software (National Institutes of Health, Bethesda, MD, USA).

### 2.6. Determination of Glucose, Lactate, Glutamine, and Glutamate

The concentration of metabolites in the supernatant from cellular cultures was measured using a biochemistry analyzer (YSI 2900, Yellow Springs Instruments). Membranes containing specific enzymes were used for metabolite determination (d-glucose oxidase, l-glutamine oxidase, l-lactate oxidase, and l-glutamic acid oxidase). Specific standards were prepared according to the manufacturer's recommendations (Yellow Springs Instruments). The volume of evaporation after culture incubation was measured to correct the quantity of each metabolite. Total protein from whole cellular cultures was determined and used to normalize metabolites' concentration, which is reported in mmol/L∗mg protein.

### 2.7. Immunofluorescence

Cells were cultured in 4-well chamber slides (Nunc Lab-Tek; Thermo Fisher Scientific, Carlsbad, CA, USA) after incubation cells were fixed with a 4% paraformaldehyde solution for 15 min at room temperature. After rinsing with PBS, cells were blocked and permeabilized with 0.5% Triton x-100 (Research Organics) in PBS with 2% normal pig serum for 30 min at room temperature. Cells were then washed and incubated overnight at 4°C with following antibodies: HIF-1*α* (Cell Signaling Technology; # 14179S), HIF-2*α* (Novus; NB100-122), and HIF-3*α* (Novus; NBP1-03155). The detection of primary antibody was performed with an anti-Rabbit IgG-Alexa Fluor 647 secondary antibody (Jackson ImmunoResearch), and nuclei were stained with DAPI (NucBlue, Thermo Fisher Scientific). Samples were then mounted with SlowFade mountant (Thermo Fisher Scientific) and imaged in a laser scanning confocal microscope (FV-1000, Olympus). Image acquisition using the confocal microscope generated separate channels for the DAPI nuclear stain and for HIF fluorescence. The image corresponding to the blue channel (DAPI) was used to automatically generate the Regions of Interest (ROI) corresponding to the nuclei of the cells. These ROI were then exported to the green channel (HIF fluorescence), and fluorescence was quantified only in the area selected by the ROI, therefore ensuring that only nuclear HIF fluorescence was measured. Image thresholding and quantification of fluorescence were performed with FIJI (ImageJ ver. 1.52p).

### 2.8. Statistical Analysis

Statistical analysis was performed with SPSS statistical software version 20.0 (IBM SPSS). Data are presented in graphs as the median and interquartile range deviation. *p* < 0.05 was considered statistically significant (^∗^*p* < 0.05, ^∗∗^*p* < 0.01, and ^∗∗∗^*p* < 0.001). For all analyses, data normality was first verified by the Kolmogorov Smirnov test. Subsequently, parametric tests (Student's *t*) and nonparametric tests (Kruskal-Wallis) were carried out, as appropriate, and if they observed differences, the Mann–Whitney *U* test was applied.

## 3. Results

### 3.1. A High Concentration of 2-ME and DCA Reduces Growth on A459 under Hypoxic Conditions

**Figure 1:**
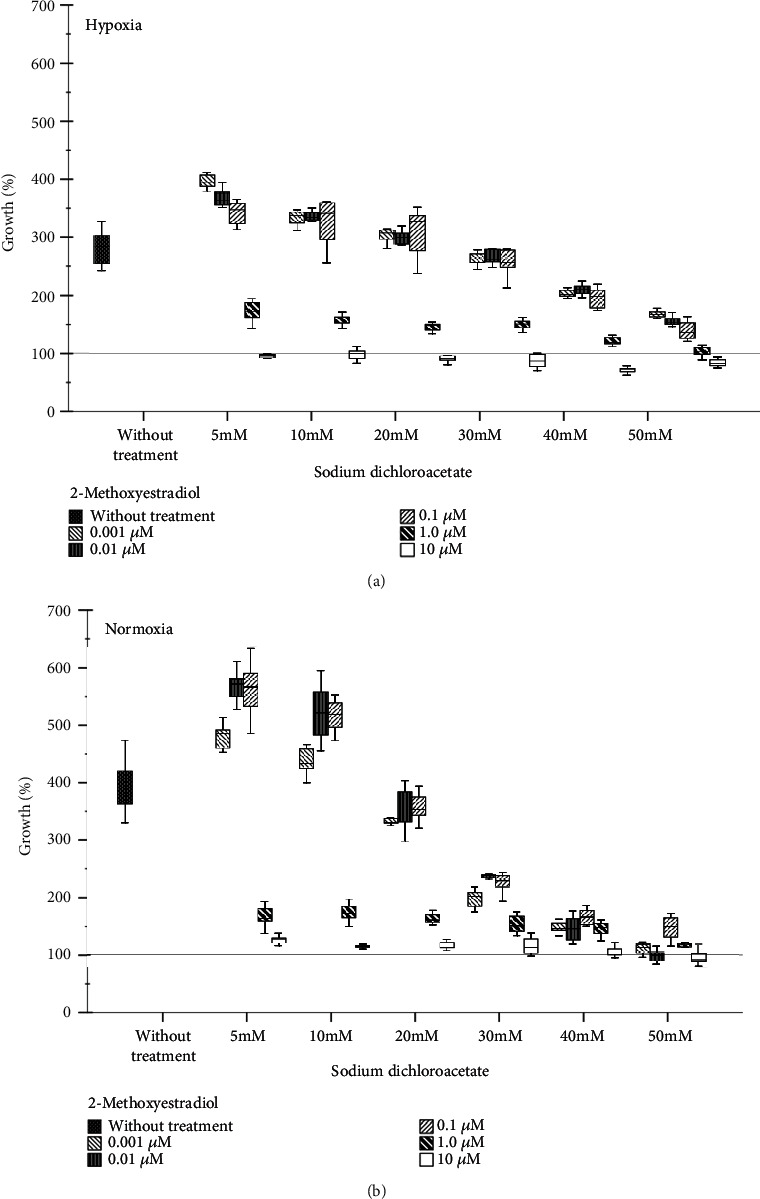
Growth rate assay under hypoxia and normoxia of A549 cells treated with different concentrations of 2-ME and DCA at 72 hours. Four independent experiments were carried out with eight wells per condition and treatment with a total of thirty-two data for each one. Hypoxia (a) and normoxia (b).

Cell growth eventually produces an oxygen demand in proportion to the number of cells; thus, normal cells facing hypoxia reduce their proliferation; however, cancer cells maintain it [10]. To determine the impact of hypoxia in the growth rate of human nonsmall lung cancer cells (A549), crystal violet assay was performed after exposing the cells for 72 hrs at 1% of oxygen concentration. Results show that hypoxia reduces the growth from 378.6% to 283.6% (*p* < 0.01) (Figure 1).

As previously reported, 2-ME is capable of inhibiting growth but not under hypoxia [8]. Therefore, in this study, 2-ME was tested in combination with DCA. In normoxia, DCA alone reduces proliferation in a dose-dependent manner, and under hypoxia, only doses above 20 mM are effective (Supplementary Figure 1). This study demonstrated that a combination of 2-methoxyestradiol (2-ME) and sodium dichloroacetate (DCA) even under hypoxic conditions stops the growth in A549 (*p* < 0.01) (Figure 1(a)). Indeed, when the concentrations of 2-ME and DCA are low, these stimulate cell growth in both conditions. Perhaps, this is because DCA in these concentrations improves its metabolism. It is important to note, under hypoxia, this combination requires higher concentrations to have an effect.

### 3.2. Hypoxia and 2-ME Inhibit Cell Migration

To test the effect of this combination of drugs on cell migration, wound healing assay was used, A549 cells were exposed to hypoxia, and the effect of each reagent was assessed.

**Figure 2:**
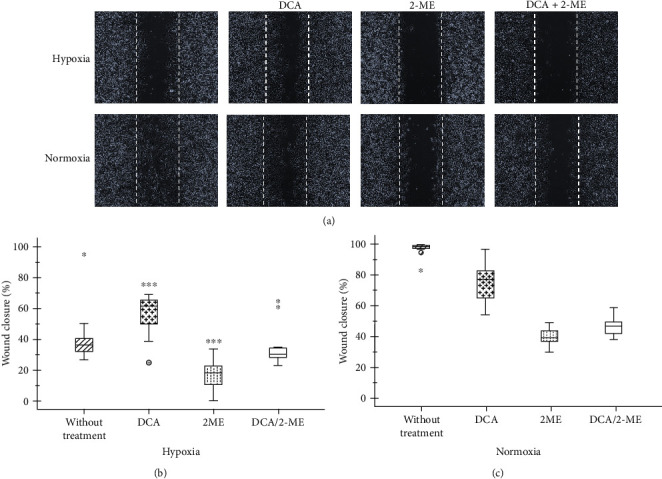
Wound healing assay under hypoxia of A549 cells treated with of 2-ME and/or DCA. (a) Representative images of wound healing in hypoxic and normoxic cells with 2-ME (10 *μ*M), DCA (40 mM), and in combination 2-ME/DCA. The graphs show the percentage of wound closure. Two independent experiments were performed, and four areas were taken giving a total of sixteen photographs per treatment in (b) hypoxia and (c) normoxia. ^∗^*p* < 0.05, ^∗∗^*p* < 0.01, and ^∗∗∗^*p* < 0.001.

Hypoxia and 2-ME show a prominent effect, both together inhibited less than 20% migration (*p* < 0.01) (Figure 2). DCA has a double outcome which initially in normoxia can decrease while under hypoxic conditions increases migration. Suggesting that DCA induces a metabolic switch where hypoxia has less activation (*p* < 0.05) (Figures 2(a) and 2(b)). Notably, the combination has an equivalent impact than 2-ME alone in normoxia and hypoxia.

### 3.3. DCA Turns Off the Glycolytic Flux and 2-ME Reduces in Part the Glutaminolysis under Hypoxia

Metabolism of cancer cells is characterized by a boosted flux of glycolysis increasing the glucose consumption and lactate production even under aerobic conditions. To determine the effects on the metabolism, the consumption of glucose and glutamine and the production of lactate and glutamate were assessed.

**Figure 3:**
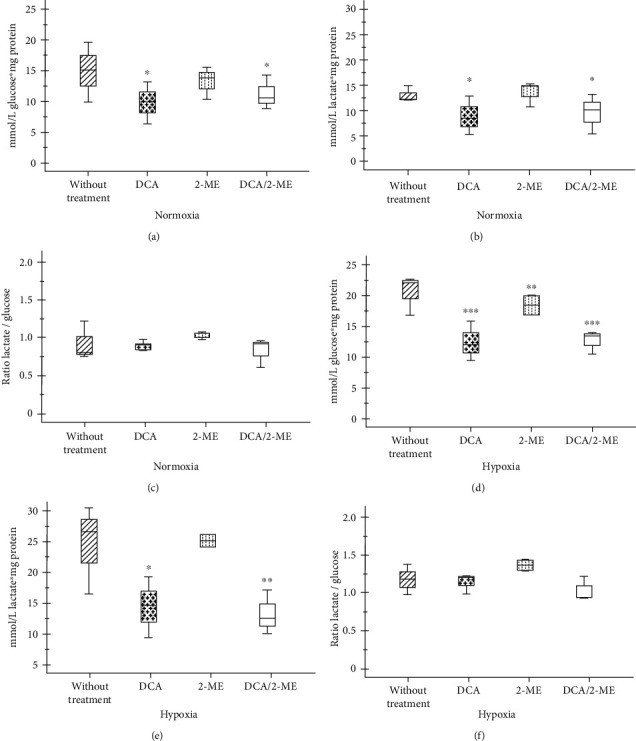
Effect of treated with 2-ME and/or DCA of A549 cells on glucose consumption and lactate production. Glucose consumption (a, d) and lactate production (b, e) and the ratio_(Lact/Gluc)_ (c, f) in normoxia and hypoxia, respectively. Three independent experiments were carried out. ^∗^*p* < 0.05, ^∗∗^*p* < 0.01, and ^∗∗∗^*p* < 0.001.

As expected, under hypoxic conditions, the cells have increased uptake of glucose and production of lactate with respect to the normoxic ones, which confirms that hypoxia favors the glycolytic metabolism with lactate production (Figure 3). In normoxia, the ratio_(Lact/Gluc)_ was 0.8; when these cells were cultured under hypoxia, the ratio_(Lact/Gluc)_ increased to 1.3 (Figures 3(c) and 3(f)). In the theoretical ratio that glucose will be used exclusively by the glycolytic path, the ratio would be 2.

As mentioned above, DCA is capable of inhibiting the PDK enzyme, DCA, or the combination of DCA/2-ME which diminished the glucose consumption and lactate production in both conditions (Figure 3). However, the ratio_(Lact/Gluc)_ of DCA treated cells did not change (Figures 3(c) and 3(f)). With DCA, carbon skeletons that are directed towards the production of lactate decrease in quantity but not in proportion. Thus, DCA alone or in combination with 2-ME diminishes the glucose quantity and glycolytic flux.

**Figure 4:**
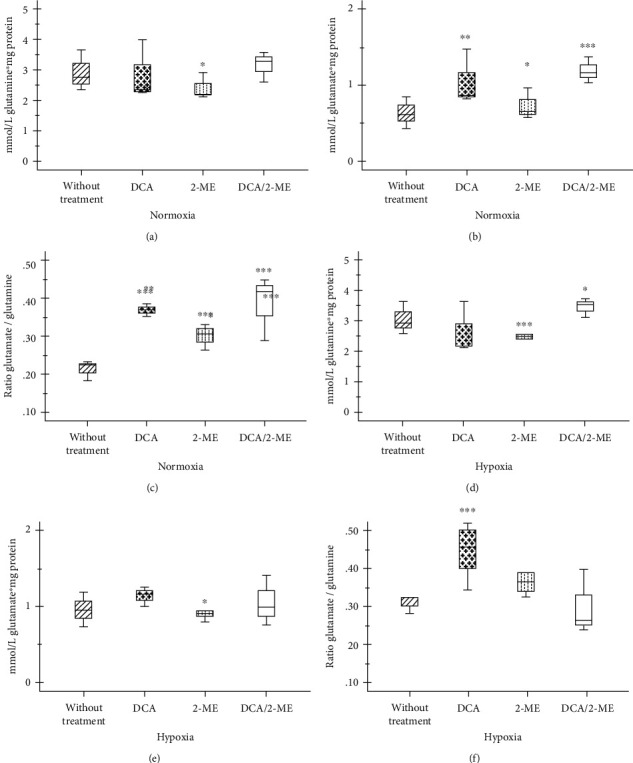
Effect of treatment with 2-ME and/or DCA of A549 cells on glutamine consumption and glutamate production. Glutamine consumption (a, d) and glutamate production (b, e) and the ratio_(Gte/Gne)_ (c, f) in normoxia and hypoxia, respectively. Three independent experiments were carried out. ^∗^*p* < 0.05, ^∗∗^*p* < 0.01, and ^∗∗∗^*p* < 0.001.

On the other hand, it has been reported that tumor cells are addicted to glutamine [11]. A549 cells consumed the same amount of glutamine under hypoxia; however, these cells increase glutamate production and the ratio_(Gte/Gne)_ (Figure 4).

Additionally, under hypoxia, 2-ME-treated cells diminished glutamine consumption (Figures 4(c) and 4(f)). In this case, the combination (2-ME 10 *μ*M/DCA 40 mM) shows an opposite effect to each other (Figure 4). Thus, under hypoxia, 2-ME treated cells presented less glutamate availability for glutathione (GSH) synthesis or for Krebs cycle replenishment.

### 3.4. 2-ME and DCA Combination Decreases Hypoxia Signaling by HIF-3*α*

**Figure 5:**
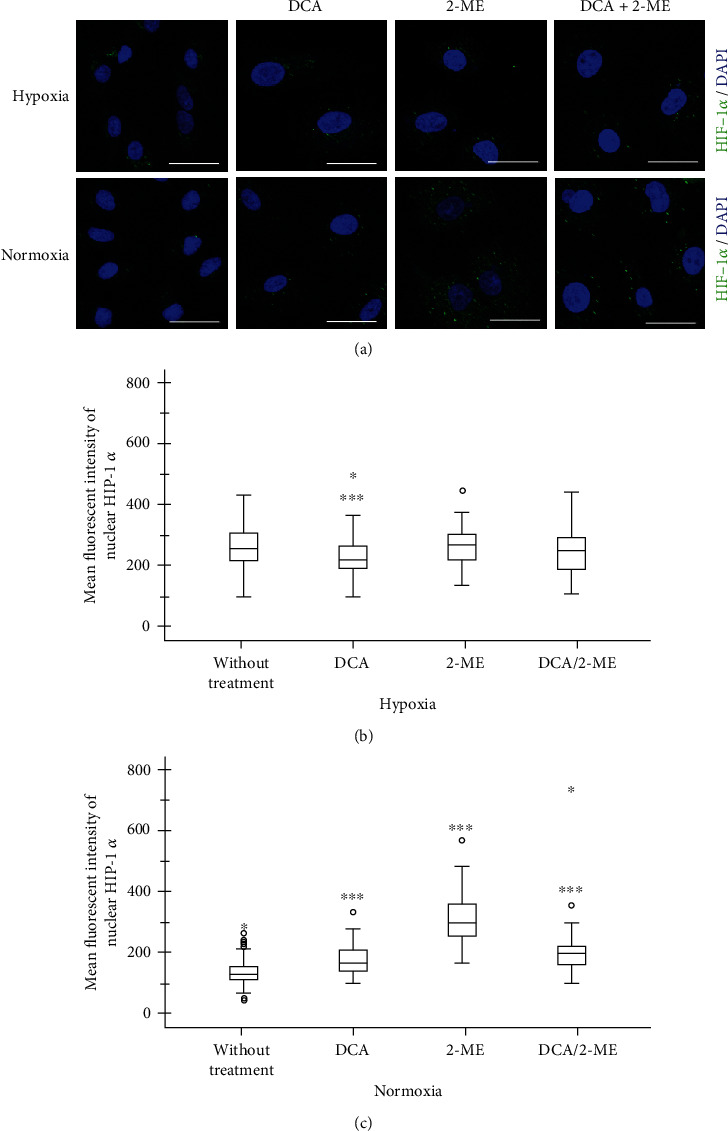
Representative micrographs of HIF-1*α* expression in A549 cells under hypoxia treated with DCA, 2-ME, or the combination. (a) The transcription factor is shown in green and nuclei in blue. 5 × 10^3^ cells per well in normoxia and 8 × 10^3^ cells per well in hypoxia were cultured in four-well chamber slides for 72 h with their respective treatments: DCA 40 mM, 2-ME 10 *μ*M, DCA 40 mM/2-ME 10 *μ*M combination, and control without treatment (2% FBS). Quantification of fluorescence was performed in nuclei: (b) hypoxia and (c) normoxia. *n* > 50 cells. ^∗^*p* < 0.05, ^∗∗^*p* < 0.01, and ^∗∗∗^*p* < 0.001.

**Figure 6:**
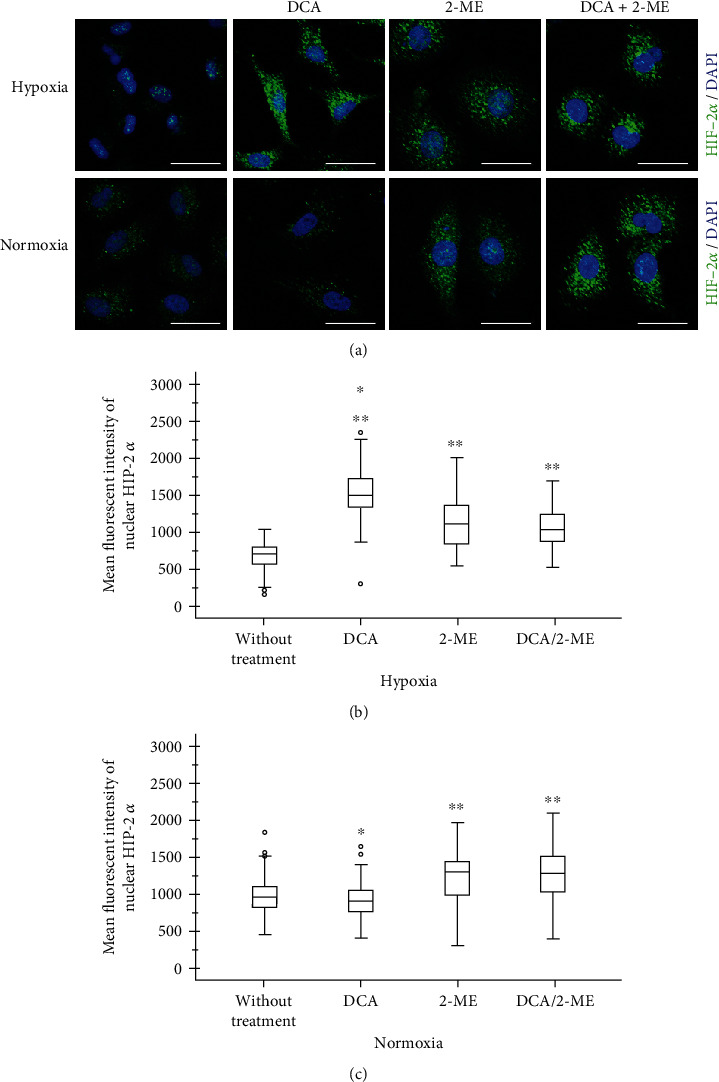
Representative micrographs of HIF-2*α* expression in A549 cells under hypoxia treated with DCA, 2-ME, or the combination. (a) The transcription factor is shown in green and nuclei in blue. 5 × 10^3^ cells per well in normoxia and 8 × 10^3^ cells per well in hypoxia were cultured in four-well chamber slides for 72 h with their respective treatments: DCA 40 mM, 2-ME 10 *μ*M, DCA 40 mM/2-ME 10 *μ*M combination, and control without treatment (2% FBS). Quantification of fluorescence was performed in nuclei: (b) hypoxia and (c) normoxia. *n* > 50 cells. ^∗^*p* < 0.05, ^∗∗^*p* < 0.01, and ^∗∗∗^*p* < 0.001.

**Figure 7:**
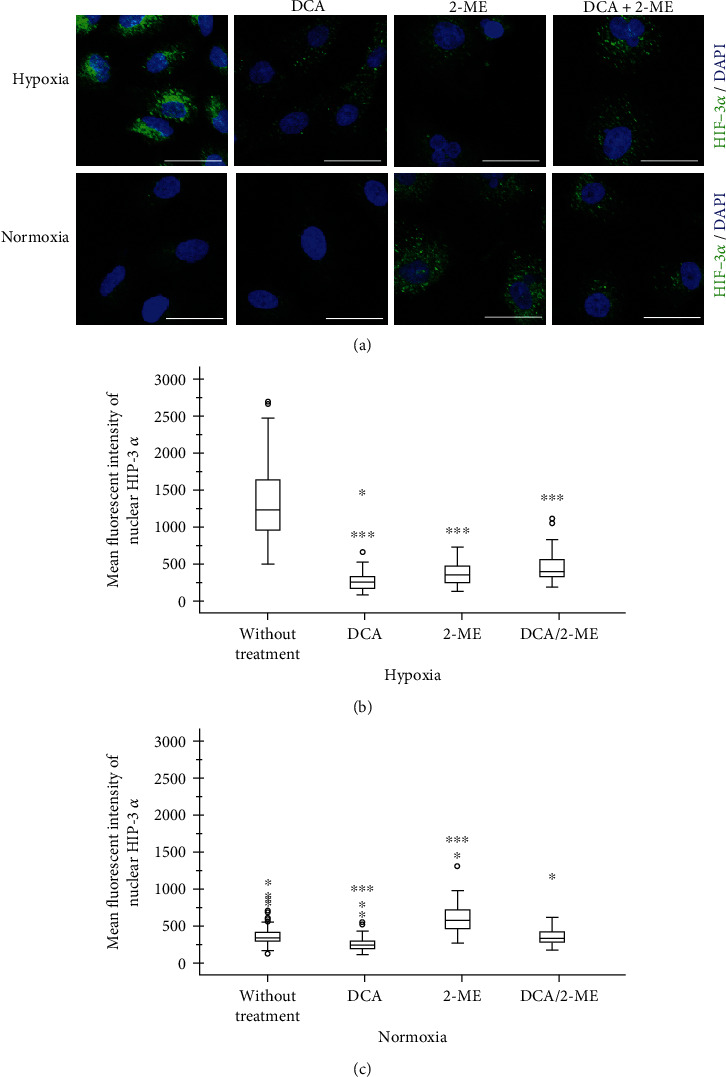
Representative micrographs of HIF-3*α* expression in A549 cells under hypoxia treated with DCA, 2-ME, or the combination. (a) The transcription factor is shown in green and nuclei in blue. 5 × 10^3^ cells per well in normoxia and 8 × 10^3^ cells per well in hypoxia were cultured in four-well chamber slides for 72 h with their respective treatments: DCA 40 mM, 2-ME 10 *μ*M, DCA 40 mM/2-ME 10 *μ*M combination, and control without treatment (2% FBS). Quantification of fluorescence was performed in nuclei: (b) hypoxia and (c) normoxia. *n* > 50 cells. ^∗^*p* < 0.05, ^∗∗^*p* < 0.01, and ^∗∗∗^*p* < 0.001.

In order to evaluate the role of alpha subunits after treatment with the combination of drugs on A549 cells, the expression of HIF-1*α*, HIF-2*α*, and HIF-3*α* was assessed by immunofluorescence and quantified according to its nuclear localization. As anticipated, hypoxia increases the expression of HIF-1*α* and HIF-3*α* significantly (Figures 5 and 6). By contrast, HIF-2*α* showed an accumulation in normoxia, and under hypoxia, it had an exclusive location within the nucleus (Figure 7). This effect could be a particular alteration on hypoxia signaling by HIF-2*α*.

Regarding the combination of these drugs on HIF signaling, the most remarkable finding of this study is the fact that under hypoxia the combination showed an effect by reducing HIF-3*α*. After treatment with the drug combination, the expression level of HIF-3*α* was reverted to levels comparable to those of normoxia (Figure 6). Importantly, when each reagent was evaluated separately, DCA showed an inhibition of HIF-3*α* and a modest effect in HIF-1*α* (Figures 5 and 6), while 2-ME increased HIF-1*α*, HIF-2*α*, and HIF-3*α* in normoxia. This result indicates that 2-ME has an effect on HIF signaling independently of oxygen levels. In the case of HIF-2*α*, it had similar results independently of oxygen levels and the treatment 2-ME or DCA increase its levels under hypoxia (Figure 7).

## 4. Discussion

Lung cancer is considered a public health problem due to its high mortality and the scarcity of effective therapeutic resources [12]. A hypoxic microenvironment induces metabolic changes that could itself promote the phenotype of cancer and resistance to treatments [13]. Hypoxia response, which includes signaling pathways and gene expression changes regulated by HIFs, involves a distinct perspective that requires a comprehensive understanding of its relationships in cancer biology [14]. So, assuming this, we focused on this metabolic alteration induced by hypoxia as a driver for cancer progression. Our work demonstrates that a combination of 2-ME (10 *μ*M) and DCA (40 mM) under hypoxia showed an inhibitory effect of cell growth, migration, reduced glycolysis, and HIF-3*α* expression as a potential therapy tested on A549 cells.

Cancer aggressiveness is depicted by growth and metastasis, which is associated with migration. The effect of 2-ME on growth and migration is predominant, since 2-ME has already been reported to be able to induce the arrest of the cell cycle [15] and as an inhibitor of tubulin polymerization [16]. However, a hypoxic environment diminishes its effect. Indeed, in a previous work, we report that 2-ME reduces growth and induces apoptosis in A459 under normoxic conditions, but not under hypoxia [8]. Furthermore, it is important to emphasize that the hypoxic environment has a biphasic behavior; it reduces growth and migration until a certain threshold, after which changes induced by this environment increase resistance to the treatments. For example, this combination (2-ME and DCA) under hypoxia requires a higher dose to reduce A549 growth. A limitation of this study was the lack of experiments in the absence of glucose or glutamine to establish if this alteration is due to specific nutrients. On the other hand, regarding DCA and cell migration, the effect is not clear since it presents a different direction according to normoxia or hypoxia. Despite these contradictory effects, the important result about this combination is that it reduced growth and migration robustly.

Tumor cells can redirect metabolic pathways to assist the malignant processes [17]. The typical example is “the Warburg effect” or aerobic glycolysis. Glucose consumption and lactate production induced by hypoxia or by the Warburg effect are reversed by DCA, and these results agree with several studies [18, 19]. In addition, a decrease in lactate production also reduces acidosis, which is involved in tumor progression [20].

This metabolic shift (glycolysis) matches with glutaminolysis which provides intermediates for the TCA cycle [17]. Although both drugs affected glutamine metabolism, only 2-ME significantly decreased glutamine uptake under hypoxia, while in the combination, this effect was not observed. Therefore, the possibility of another drug that could inhibit glutaminolysis simultaneously is open as an important step in metabolic treatment [21].

A major mechanism for adaptation of cancer cells to the hypoxic microenvironment is HIF signaling [22]. Regarding this relevance, these experiments highlight the vast concentrations of HIF-2*α* that suggest a constitutive activation independent of oxygen sensing, also reported by Sato et al. [23]. Similar evidence has been reported by mutations in oncogenes that can go in the direction of HIF-1*α* signaling as mTOR, VHL, and others [24]. In response to treatment with 2-ME and DCA, the principal result is the decrease of HIF-3*α* expression under hypoxia. Importantly, DCA inhibits HIF-1*α* and HIF-3*α* under hypoxia and HIF-2*α* in normoxia. The mechanism by which DCA reduces HIF signaling has not been explored. Among the possibilities, it may be the result of recovering mitochondrial function, but mitochondria function was not determined [25]. In addition to this, cancer cells can evade controls within the cell by mTOR and AMPK complexes, in which complexes regulate stress due to oxygen depletion and periods of intense metabolism [26]. Another possibility is that DCA treatment alleviates some of this energy or acidic stress.

In the case of HIF-3*α*, the direction of their regulations is not clear, since in preceding descriptions it is considered a negative regulator of HIF-1*α* and HIF-2*α* by the lack of transactivation domain [27]. HIF-3*α* is the less-studied factor, and has several isoforms reported; the mouse antibody used in this work is against a peptide sequence close to 575-600aa, so six human isoforms are aligned in a very similar way, but this antibody does not detect the HIF-3*α* 4 isoform, so the quantification is not for all of the isoforms. HIF-3*α* isoform is also involved in cancer biology [28].

Nonetheless, the effect of each reagent requires further research. In view of the fact that 2-ME induces an increase of HIF-1*α*, HIF-2*α*, and HIF-3*α* with oxygen available, this effect is similar to that observed in HIF-2*α*, but as they were assessed together and did not show an effect probably is by a different pathway.

This is the first study that integrates HIFs on A549 under hypoxic conditions; it only makes sense because HIF participation is diverse and complex. For example, we show this complex relationship between the different members of HIF in idiopathic pulmonary fibrosis, where lack of HIF-3*α* in HIF response, by its particular hypermethylation, is associated with an increase in cell differentiation [29]. Beyond these particularities, alterations in hypoxia-response represent the correlation that exists in both diseases [30].

Hypoxia has repercussions that are not exclusive to metabolism or HIF signaling; it can also affect genomic instability, telomerase expression, etc. that can also promote an aggressive phenotype. However, considering the mechanisms of the tumor response to hypoxia, HIF signaling and especially the altered metabolic pathways give us the guideline to establish new strategies to eradicate cancer cells, which could improve the effectiveness of current therapies against cancer [31, 32].

## 5. Conclusion

In summary, hypoxia reduces the effect of treatments. It is important to highlight that under hypoxic conditions this combination (2-ME 10 *μ*M DCA 40 mM) is able to stop the growth, as well as inhibit HIF-3*α*. In addition, the effect that has been reported for each one was maintained; migration was reduced using 2-ME, while DCA shows a decrease in glycolysis. Therefore, both may work in therapy perhaps with a third component that can reduce HIF-2*α*.

## Data Availability

The data that support the findings of this study are openly available.

## Supplementary Materials

Supplementary Materials Supplementary Figure 1: growth rate assay under hypoxia and normoxia of A549 cells treated with different concentrations of DCA at 72 hours. Three independent experiments were carried out with eight wells per condition. ^*^*p* <0.05, ^**^*p* <0.01, and ^***^*p* <0.001.
