# Supplementary figures and images for: Antitumor Therapy under Hypoxic Microenvironment by the Combination of 2-Methoxyestradiol and Sodium Dichloroacetate on Human Non-Small-Cell Lung Cancer
Source: Oxid Med Cell Longev. 2020 Oct 23;2020:3176375. doi: 10.1155/2020/3176375 (PMC7603622; doi:10.1155/2020/3176375)

## Slide 1
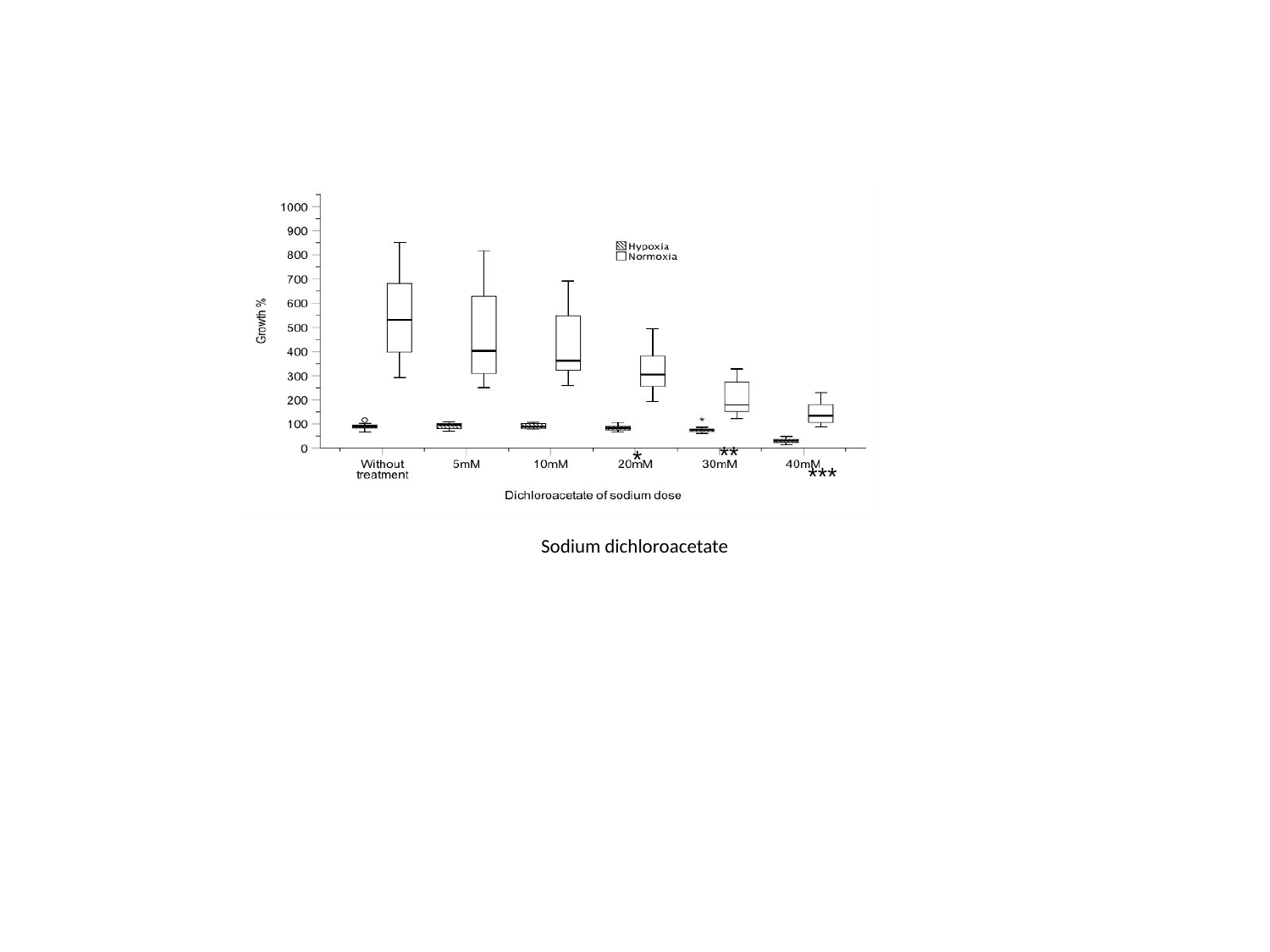

Sodium dichloroacetate
 **
 *
 ***

Supplement: Supplementary Materials — Supplementary Figure 1: growth rate assay under hypoxia and normoxia of A549 cells treated with different concentrations of DCA at 72 hours. Three independent experiments were carried out with eight wells per condition. *p <0.05, **p <0.01, and ***p <0.001. [file 3176375.f1.pptx]
